# Evolutionary and physiological arguments for the existence of a circalunar clock in humans

**DOI:** 10.1186/s12862-026-02550-8

**Published:** 2026-06-30

**Authors:** Charlotte Helfrich-Förster, Thomas A. Wehr

**Affiliations:** 1 Neurobiology and Genetics, Biocenter, Am Hubland, Julius-Maximilians-University of Würzburg, 97074 Würzburg, Germany; 2 Intramural Research Program, National Institute of Mental Health, Bethesda, MD, USA

**Keywords:** Circalunar clocks, Moonlight, Sleep, Mood, Menstrual cycle, Entrainment range, Artificial light at night, Relative coordination

## Abstract

Life on Earth evolved in the presence of recurring environmental cycles, namely 12.4-hour tidal cycles, 24-hour day-night cycles, 14.77-day semilunar cycles, 29.5-day lunar cycles, and 1-year annual cycles. In anticipation of predictable conditions associated with these geophysical cycles, organisms have evolved corresponding endogenous time-keeping mechanisms—so-called biological clocks. These clocks oscillate with periods that closely match the periods of the environmental cycles and are therefore called circatidal, circadian, circasemilunar, circalunar, and circannual clocks. While circadian clocks are best studied, and circannual clocks are known to exist in several species, the existence of circatidal, circasemilunar and circalunar clocks is often questioned in terrestrial species. The role of semilunar and lunar rhythms in the reproduction of marine organisms is well known, and they have been shown to rely on circa(semi)lunar clocks in several cases. As life originated in the sea, circa(semi)lunar clocks are likely to be ancient timing mechanisms, which may also be present in terrestrial animals, including humans. Increasing evidence suggests that physiology, metabolism and behaviors including reproduction follow (semi)lunar rhythms in humans and many other animals. Here, we present an overview of (semi)lunar rhythms in animals, with evidence that some of the rhythms are governed by circa(semi)lunar clocks, including those in humans. We compare the properties of circadian clocks with those of circa(semi)lunar clocks, including their entrainment by environmental light cycles - daily light/dark cycles in the former case and monthly full moon/new moon cycles in the latter case, and we examine their putative selective advantages. Finally, we discuss the negative effects of artificial light at night on the entrainment of the putative human circa(semi)lunar clock. Based on all of these observations, we hypothesize that humans possess a circa(semi)lunar clock that was mainly entrained by the environmental moonlight cycles before the introduction of artificial light.

## Background

Life on Earth evolved around 3.7 to 3.8 billion years ago, more than a billion years after the Moon was formed by the collision of a Mars-sized celestial body with the protoplanet Earth [1, 2]. This means that life developed not only under the influence of the Sun, but also under the influence of the Moon. As life developed, the Moon’s influence on Earth was even greater than it is today, as the distance between Earth and the Moon is constantly increasing (3.8 cm per year = 144,400 km since the emergence of life = about one third of the Moon’s current distance from Earth) [3]. But even today, the moon still has a major influence on Earth: it stabilizes the Earth’s axial tilt, creates the tides in the oceans and provides significant night-time illumination.

Due to the rotation of the Earth around its own axis, its orbiting around the Sun and the orbiting of the Moon around the Earth, the environment on Earth is highly rhythmic. There are recurring 12.4-hour tidal cycles, 24-hour cycles of night and day, 14.77-day semilunar cycles, 29.5-day lunar cycles, and 1-year annual cycles. To anticipate these predictable rhythms, organisms have evolved corresponding endogenous time-keeping mechanisms—so-called biological clocks. These endogenous clocks oscillate with periods that closely match the periods of the different environmental cycles, meaning that their periods are circa 12.4 h, circa 24 h, circa 14.77 days, circa 29.5 days, and circa 1 year long. Therefore, the different biological clocks are called circatidal, circadian, circasemilunar, circalunar and circannual clocks. Normally, these endogenous biological clocks run in synchrony with the ambient rhythms, which are called Zeitgebers (time givers).

Endogenous biological clocks are precisely defined [4–6]: (1) They continue to run even in the absence of Zeitgeber cycles, and under such circumstances they “free-run” with their endogenous period. (2) They are able to “entrain” to Zeitgeber cycles such as light-dark or temperature cycles. Entrainment is an active synchronization to the Zeitgeber cycles. During entrainment, endogenous clocks maintain a stable phase relationship with the Zeitgeber cycle. This phase relationship depends on their endogenous period and their light sensitivity. (3) Endogenous clocks are temperature-compensated, which means that their endogenous period does not depend on the ambient temperature but remains constant, at least within certain range of physiologically relevant temperatures. (4) Endogenous clocks have a limited range of entrainment, which means that they cannot entrain to Zeitgeber periods that are too far from their endogenous period. At the limits of their entrainment range, they can show relative coordination, meaning that they alternate between following the Zeitgeber and free-running with their endogenous period. When the Zeitgeber period is too far from the endogenous period, they ignore the Zeitgeber and free-run with their endogenous period all the time. (5) Endogenous clocks can also follow phase shifts of the Zeitgeber, but they need a certain number of cycles (transients) until they are re-entrained to the shifted Zeitgeber cycle (have the original phase relationship with the Zeitgeber cycle).

Circadian clocks meet all the above criteria, and this has been shown for many organisms. However, the situation is different for the circatidal, circasemilunar, circalunar and circannual clocks, the properties of which have only been tested in few, selected species. Therefore, circatidal, circasemilunar, circalunar and circannual clocks are still ‘nature’s enigmatic clocks’ [7]. Here, we will focus on lunar rhythms and lunar clocks, but we will see that they are not completely independent of circadian, circatidal and circannual clocks - and vice versa. First, we will describe the geophysical rhythms the moon imposes on earth and give an overview of lunar biological rhythms, followed by evidence that at least some of them are controlled by a circalunar clock. Finally, we discuss the pros and cons of the existence of a human circalunar clock and the impact of artificial light on it.

### Rhythmic geophysical influences of the Moon on Earth

**Fig. 1:**
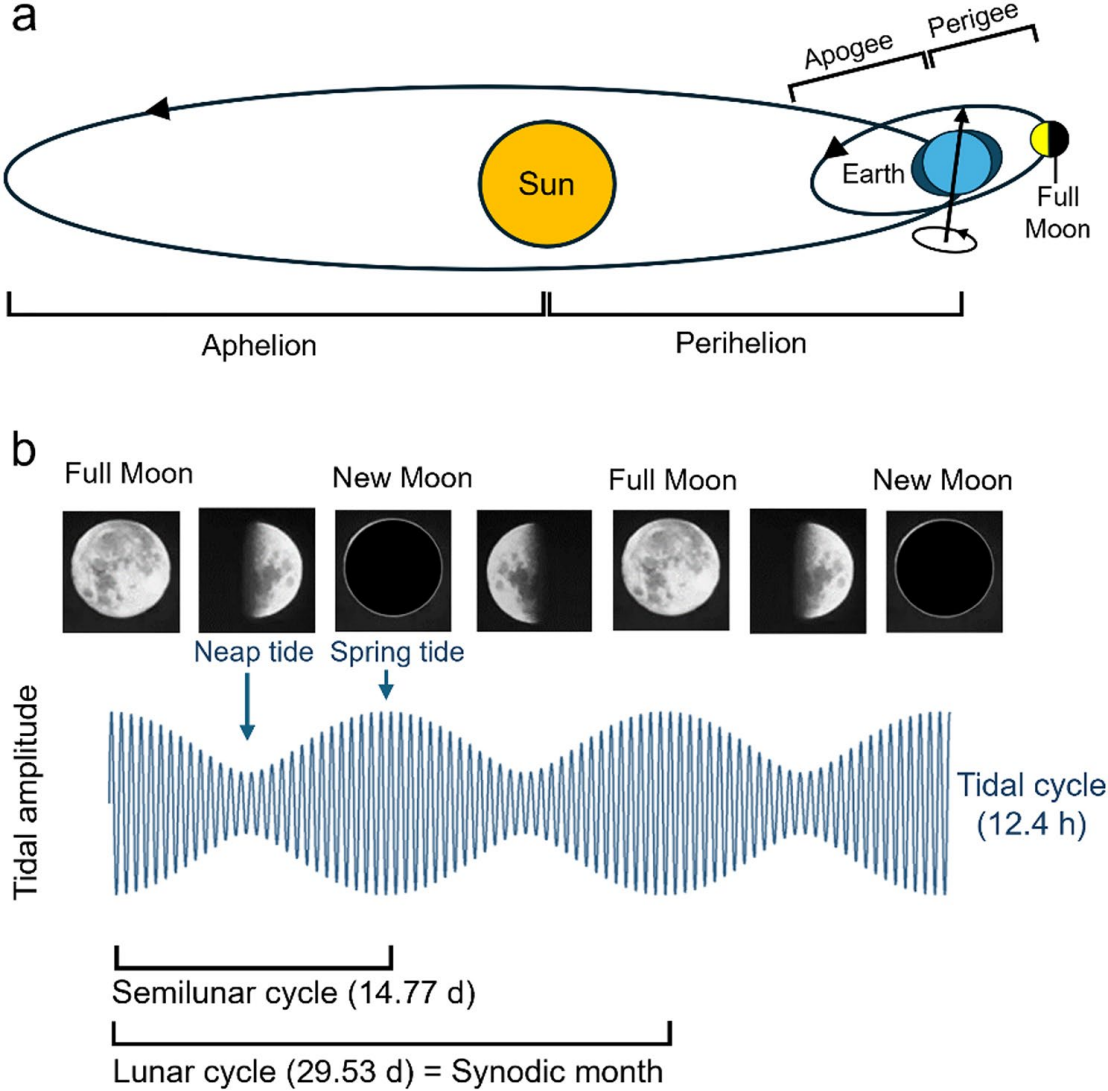
Simplified geophysical interactions between the Sun, Moon, and Earth. **a**. Schematic, not-to-scale representation of the relationship between the Sun, Earth, and Moon on January 3, 2026, when there was a full Moon, the Earth was closest to the Sun (at its perihelion) and the Moon was close to the Earth (almost at its perigee). **b**. Relationship between the lunar cycle and the tidal cycle. Tides are caused by the Earth’s rotation on its axis and repeat every 12.4 h. The period of the tidal cycles is 0.4 h longer than would be expected based on the Earth’s rotational speed, which is due to the fact that the Moon orbits the Earth in the same direction in which the Earth rotates on its axis. Similarly, the period of the lunar cycle (synodic month) of 29.53 days is longer than the time it takes for the Moon to orbit the Earth (27.32 days) because the Earth orbits the Sun in the same direction as the Moon orbits the Earth. Therefore, it takes 2.21 days longer for the Sun, Earth, and Moon to line up again and for the next full or new moon to occur. The semilunar cycle marks the time (14.77 d) between two consecutive spring tides, which occur at both the full Moon and the new Moon

The Moon causes rhythms in nighttime illumination and gravity on Earth (Fig. 1) (for details see [8]). The best-known lunar cycle (or lunar month) is the synodic month, which represents the cycle from one full Moon to the next full Moon. The synodic month has a period of 29.53 days and is caused by the Moon’s orbit around the Earth and the Earth’s orbit around the Sun. At full Moon and new Moon, the Sun, Earth, and Moon are aligned (in syzygy), so that at full Moon the “front” of the Moon is completely illuminated by the Sun (the Sun and Moon are on opposite sides of the Earth) (Fig. 1a), and at new Moon the “back” of the Moon is illuminated by the Sun, which means that the Moon is barely or not at all visible from Earth (the Sun and Moon are on the same side relative to the Earth). During a full Moon and new Moon, when the Sun, Earth, and Moon are in syzygy, the gravitational forces of the Moon and Sun add up on the Earth, leading to spring tides, while during a half Moon we have neap tides (Fig. 1b). Therefore, the sequence of spring tides and neap tides repeats every 14.77 days, a cycle that is called semilunar month.

For several reasons the gravitational effects of the Moon on Earth are much more complex than just described. First, the Moon’s orbit around Earth is elliptical and Earth is not at the center of this ellipse, meaning that the Moon oscillates between a large (apogee) and a small (perigee) distance from Earth. The duration of this cycle is 27.55 days, and the corresponding month is called the anomalistic month. During the perigee, the gravitational forces of the Moon are greater than during the apogee (see [9, 10]). Second, the plane of the Earth’s equator is inclined relative to the Earth’s orbit around the Sun, which means that the Moon alternates between a high and a low position on the Earth’s horizon, which also affects gravity, but also to a certain extent the nighttime illumination. This cycle is called the tropical month and has a duration of 27.32 days. Third, the Earth’s orbit around the Sun is also elliptical and the Sun is not exactly at the center of the orbit, which means that once a year (always at the beginning of January), the Earth reaches its closest approach to the Sun (perihelion), while at the beginning of July, it is at its greatest distance from the Sun (aphelion). Since the gravitational forces of the Sun add to those of the Moon, the highest spring tides on Earth (so-called king tides) are observed in January [11]. When the Moon is additionally at its perigee as depicted in Fig. 1a, these king tides are exceptionally high, but such a situation occurs rarely (about once every > 100 years).

In this review, we will focus on the synodic month, and when we talk about lunar rhythms and circalunar clocks, we are referring to rhythms with a period of approximately 29.53 days. Nevertheless, we will later return to the anomalistic and tropical months and the perihelion when discussing lunar rhythms in humans.

### Lunar and semilunar rhythms in marine and terrestrial species

Lunar rhythms in sexual maturation and reproduction can be observed in many marine organisms. Some organisms show semilunar instead of lunar rhythms, meaning that they can mature or reproduce during the full and new moon, so that their maturation or reproduction can be observed every 14.77 days instead of every 29.53 days. However, this does not mean that every individual reproduces with a period of 14.77 days, it rather concerns the entire population of this species. Furthermore, semilunar population rhythms can change to lunar rhythms depending on the light conditions [12–14]. Here, we will treat semilunar rhythms as lunar rhythms, which can be linked to either the full or the new moon.

A typical example for a semilunar rhythm is the life cycle of marine midges, like that of *Clunio marinus* [15]. The larvae of *C. marinus* live in the lower intertidal zone, which only becomes dry during spring tides. Therefore, the adult midges eclose synchronously from their pupa at the lowest water level during spring tides at full and new Moon. This event is controlled by closely interacting lunar and circadian clocks, in which the lunar clock determines the time of larval pupation, and the circadian clock the time of eclosion of the adult from the pupa [12]. Once eclosed, the adults copulate and subsequently lay eggs, until they die with the next incoming tide. Under optimal conditions the life cycle lasts about 15 days but, when necessary, the larval stage can be prolonged until the next spring tide. Furthermore, depending on local tide conditions, certain *C. marinus* populations diverged and show lunar rhythms with eclosion only at full Moons or only at new Moons [14, 16].

Many marine species show semilunar rhythms in swimming activity and reproduction (summarized in [17, 18]). For example, several algae have a semilunar rhythm in gamete release, some mollusks spawn at the full and new Moon, and some crustaceans show semilunar rhythms in egg laying or larval hatching. Furthermore, spawning with semilunar periodicity is a common reproductive strategy in many teleost fish species living in the intertidal zone such as the pacific grunion, *Leuresthes tenuis* [19], the mummichog, *Fundulus heteroclitus* [20, 21], the Gulf killifish, *Fundulus grandis* [22], the grass puffers, *Takifugu niphobles* and *Takifugu alboplumbeus* [23, 24], the mudskipper (*Boleophthalmus pectinirostris*) [25], and many others. Of these, the pacific grunion is probably the most famous example, because the animals leave the water to spawn on land, with females burying themselves tail-first into wet sand to lay eggs while males wrap around them to fertilize [26], a spectacle that happens during their breeding season from March through September in the middle of the night and that became a popular attraction to people in Southern California. This kind of semilunar “amphibian” life during the breeding season is also typical for several of the other fish species noted above.

Semilunar spawning rhythms in reproduction are not restricted to marine animals but also present in amphibians that live in fresh water without any association with the tides (reviewed in [18]). For example, newts (*Lissotriton vulgaris* and *Lissotriton helveticus*) arrive at their breeding sites most frequently around the full and new Moon [27]. Other fish and amphibian species show lunar spawning rhythms occurring most frequently around the full moon [27–32].

The most spectacular lunar rhythms have again been described for marine organisms [33, 34]. Of these, the synchronous spawning of more than 30 coral species on the Great Barrier Reef in accordance with the lunar phase are probably the best known [35]. However, there are many other fascinating examples, such as reproduction of the palolo worms (*Eunice viridis*), which shed their rear body segments that are filled with eggs and sperm once a year, in the third quarter of the moon in October or November [36, 37]. Similarly fascinating is the synchronized swarming for mating in Atlantic fireworms, *Odontosyllis spec*., in which the females rise to the surface and release eggs while emitting a brightly glowing substance. The males respond to the flash of light and fertilize the eggs. This spectacle takes place on the night before the fourth quarter moon. Equally spectacular is the mating dance of the marine bristle worm *Platynereis dumerilli* from the Mediterranean, which takes place during the darkest phase of the night just after the full Moon and ends with the entire population of worms releasing their eggs and sperm [13, 38]. Depending on the worm population and the light conditions, swarming can also be semilunar and occur after the full and new Moon [13, 39, 40].

Most aquatic and amphibian organisms that reproduce in a lunar or semilunar cycle depend on external fertilization, in which the gonadal products of all individuals are released into the open water. A perfect synchronization between males and females is essential for successful reproduction.

Other species that undergo internal fertilization, such as *Clunio* spec., have a very short lifespan as adults and use the moon to synchronize their development so that males and females emerge at the same time and can reproduce successfully. This also applies to some insects that develop in freshwater. For example, African mayflies (*Povilla adusta*) emerge synchronously from lakes or rivers during a full moon, swarm, mate, and lay their eggs before dying [41]. African chironomids, *Tanytarsus balteatus*, behave similarly, but they emerge around the new moon [42].

However, there are also terrestrial species with internal fertilization that have a longer lifespan but still only meet and mate during a specific moon phase. This may be related to the release of fertilized eggs into the sea during spring tides to give the offspring the best chance of survival. For example, the giant red crabs of Christmas Island migrate from the forest to the coast once a year to mate and eventually release their fertilized eggs into the sea during a specific moon phase. Similarly, mangrove fiddler crabs (*Ucides cordatus*) search for mates during the full Moon or new Moon and choose the Moon phase according to the spring tides with the highest amplitude to increase the chances of survival of the larvae after synchronous release into the water [43]. The same applies to Japanese mountain crabs, *Sesarma hematochair* [44, 45].

Even long-lived vertebrate species that do not depend on the timely release of eggs into the water synchronize their reproduction with the lunar or semilunar cycle (reviewed by [18]). Well-known examples include sea turtles, which lay their eggs in burrows on sandy beaches, preferably around the full moon and new moon or during other specific phases of the lunar cycle, depending on the species [46, 47]. The hatchlings emerge again during specific lunar phases when the tides are high, which increases their chances of survival on their way to the sea.

Not related to the tides are the lunar calling and breeding behavior of birds (reviewed by [18]). The vocalizations of several nocturnal bird species, such as certain owls and nightjars is significantly higher during the full Moon as compared to the new Moon [48, 49], and primarily diurnal African houbara bustards perform nocturnal courtship displays exclusively during a full moon [50]. Furthermore, several species of nightjars synchronize their breeding cycle with the lunar cycle in such a way that the highest energy requirements of the brood coincide with the full Moon, which allows the adults to be most active and successful in catching prey [51–53]. Interestingly, the waxing moon even significantly increased the fertility of hens in a commercial breeding farm in Ecuador [54], suggesting that the lunar cycle affects breeding success in many birds. For mammals it is known that several species prefer to mate during a specific lunar phase. For example, wild Eurasian badgers (*Meles meles*) mate around the new moon [55], and macaques appear to have their fertile phases during the new moon [56]. Similarly, goats and horses show a higher mating behavior during new Moon [57, 58]. This may represent a selective advantage, as the likelihood of being spotted by diurnal predators is lowest at this time. In contrast, mountain gorillas, which have no natural enemies, appear to be most fertile during the full moon [9, 59].

In tropical Brahman cattle from farms in Venezuela conception rates and calving frequencies were highest around the full and new Moon [60], while the calving frequency in domesticated Holstein cattle on farms in Hokkaido (Japan), peaked shortly before the full moon [61]. The lunar phase also influences ovulation, fertilization, conception rates, sperm quality, and the timing of birth in other farm animals (summarized in [62]). As for the higher birth rate around the full moon, one could argue that nocturnal predators are much earlier detectable by diurnal prey during a full moon than on dark nights. This is particularly important for larger animals, which find it harder to hide than small badgers or macaques. In fact, lions hunt mainly in the first half of the night and especially during the waning moon, when the moon rises after sunset and it is dark at the beginning of the night [63]. This may also apply to other nocturnal predators. Terrestrial ecologists have long recognized the impact of moonlight on the success of predators and, consequently, on the risk of being hunted by predators [64, 65]. Indeed, the lunar cycle has a strong impact on many behavioral traits, some of which are related to reproduction or at least influenced by it, such as foraging and feeding, habitat use, and activity. Some animals are more active during a full Moon, others during a new Moon (reviewed in [66]). However, in most cases it is not clear whether these phenomena rely on an endogenous circalunar clock. 

### Lunar rhythms in humans

Humans have used lunar calendars since ancient times, and the Moon is at the center of numerous legends, from divine love stories to modern conspiracy theories. People believe that there are more cases of psychoses, suicides, accidents, insomnia, births, and aggression during a full Moon. There is also a belief that certain phases (e.g., the waning Moon) are optimal for dieting or make surgery less risky. However, most of these myths do not stand up to objective scrutiny. Scientists in particular are very skeptical of any observations in humans related to the moon, and there are plenty of objective studies that could not find any effects of the moon on human physiology, psychology, sleep and reproduction [67–73]. Most of these studies are based on group data analyzed in aggregate, and with few exceptions, data points consisted of the dates of events that occurred once in an individual, such as birth, violent act, suicide, crisis call, emergency room visit, hospital admission, accident, post-surgical complication, or seizure. As discussed in [56], it is not possible to determine whether a cycle of any type occurred in any individual with a single data point.

**Fig. 2:**
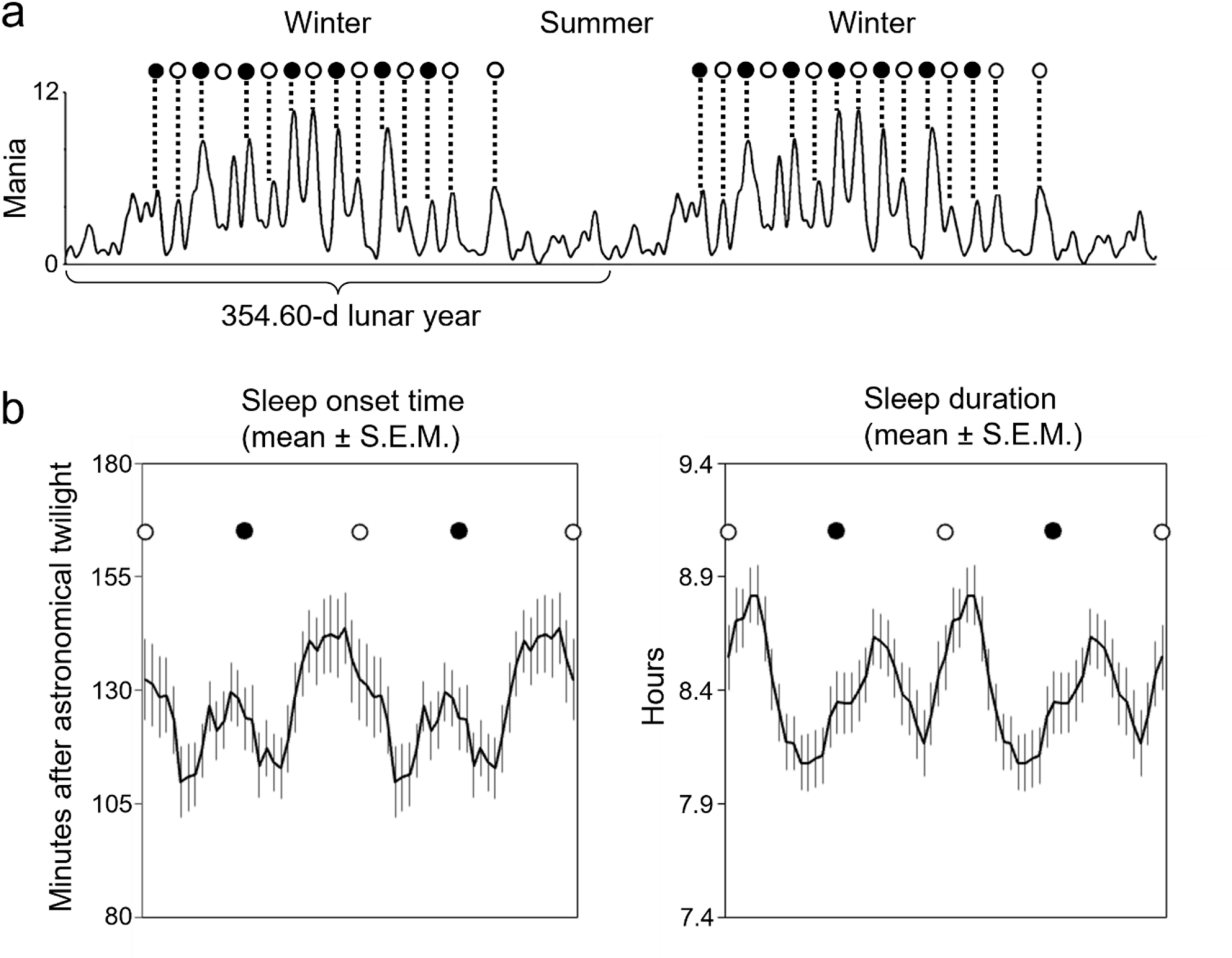
Onsets of episodes of mania in certain bipolar patients and alterations in the timing and duration of human sleep are associated with the full or new Moon. **(a)** Average 354.6-d lunar year profiles of ratings of mania in a bipolar patient. Episodes of mania reoccurred in a semilunar and annual rhythm (high in the winter and low in the summer). Data are replotted after [77]. **(b)** Sleep onset time was delayed around the full and the new Moon, and consequently sleep duration was significantly shorter at these times. Data is replotted from [79]. Note that all rhythms are double plotted for clarity

In recent years there have been increasing signs that some of the human lunar stories may be true. For example, in long-lasting observations, Thomas Wehr and David Avery found that sleep fluctuations and the alternation between mania and depression in bipolar patients coincide with the changing phases of the moon [74–77]. Figure 2a shows the recurrence of mania in a patient over a period of one year, revealing a semilunar rhythm with peaks around the full moon and new moon, as well as an annual rhythm with high mania in winter and low mania in summer (after Wehr and Avery [77]).

Human sleep also appears to be significantly influenced by the lunar cycle [78–80]. A retrospective study in the sleep laboratory revealed that humans go to bed later, sleep less, have a worse sleep quality, lower melatonin levels, and less deep slow wave sleep and EEG-delta activity around the full moon than at other times of the lunar cycle [78]. These results could not be repeated by a later sleep laboratory study with a larger number of people [67], but [79] found similar lunar effects on sleep onset and sleep duration in three groups of native Americans and a group of students living in the large light-polluted city Seattle. Most interestingly, in the latter study, sleep onset and sleep duration show a second smaller change around the new Moon (Fig. 2b) [79]. This indicates the presence of a semilunar rhythm in sleep, as observed in mania. In addition, lunar rhythms in sleep and mood respond not only to the moon’s luminance cycles but also to its gravitational cycles [77, 80]. In fact, gravitational and luminance cycles interact with each other, and the strongest synchronization with the moon occurs when they reinforce each other. Nonetheless, moonlight appears to be the strongest Zeitgeber for lunar sleep rhythms as their amplitude decreases in highly light-polluted cities [79].

**Fig. 3:**
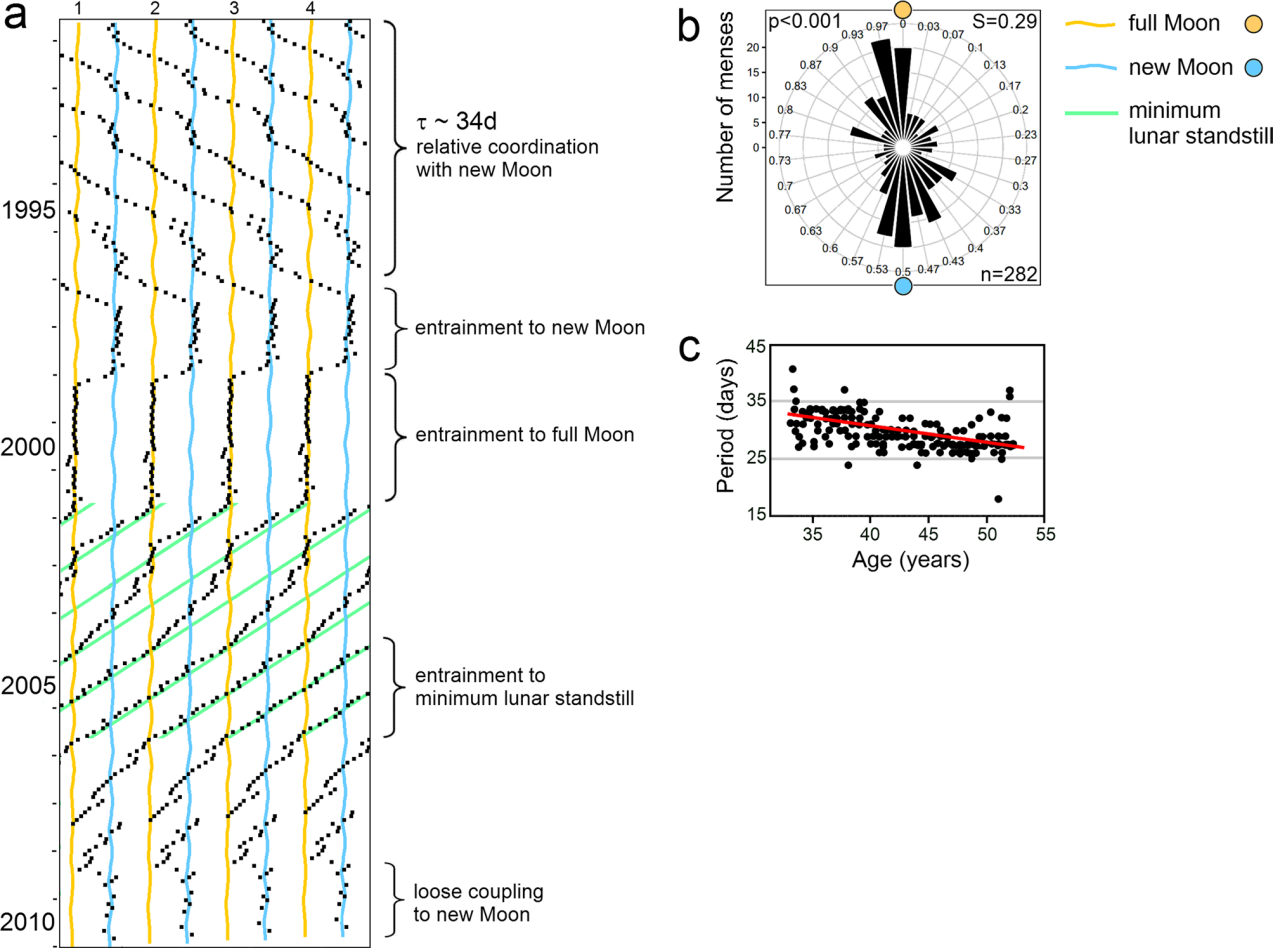
Menstrual cycle of a woman, which entrained temporarily to the new and full Moon, and to the minimum lunar standstill. **(a)** Mensogram showing menses onsets (black dots) for 19 consecutive years. To facilitate visual inspection of the course of the menstrual cycle, the plot is repeated four times with the period of the synodic month. The times of full Moons and new Moons are indicated as yellow and blue undulating lines, respectively. The times of the minimum lunar standstill are shown as green lines in the years 2001 to 2006. The data stem from a 33-year long recording (woman #1 in [9]), of which only the last 19 years are shown. **(b)** Circular plot of the distribution of menses phases throughout the synodic cycle. The synodic cycle was divided into 30 equal segments each lasting about 1 day. The number of menses onsets that occurred within a certain segment of the synodic month is plotted radially. A Rayleigh test checked for the presence of a uni- or bimodal distribution with the phases synchronized to the full/new Moon (p value in the left upper corner). The S value shown in the top right corner indicates the length of the directional vector. S values of ≥ 0.10 are regarded as highly peaked distributions. The number in the lower right corner indicates the sum of the menses. **(c)** The period of the menstrual cycle shortened over age (except for the last year before menopause, when it became irregular and longer again)

The menstrual cycle is influenced by the lunar cycle in a similar way to the sleep and mood cycles. There are reports that menstruations occur at higher frequency around the full Moon [81–83], the new Moon [84] or around full and new Moon [9, 10]. However, this was only observed in women who had a cycle length with a period close to the lunar cycle (29.53d) and in longitudinal studies of individual women (Fig. 3) (discussed in [56]), while studies that analyzed group data from hundreds to thousands of women in aggregate did not see such a relationship (e.g. [85–87]). This could be due to the nature of large-scale aggregate studies, which make it impossible to detect a temporary alignment of individual menstrual cycles with the lunar cycle.

Similar to sleep and mood cycles, the menstruation cycle can synchronize to the gravimetric cycles of the moon (Fig. 3); however, as with sleep, moonlight appears to be the strongest Zeitgeber, as synchronization decreases with increasing light-pollution [9, 10].

Overall, the observed lunar rhythms related to sleep, mood, and female fertility have several similarities. They are not particularly pronounced, but when they do occur, they can associate with the full Moon or new Moon and can therefore be regarded as semilunar. Their synchronization with the synodic lunar cycle is weak, but they can also synchronize with the gravimetric lunar cycles, with the strongest synchronization observed when luminescence and gravimetric cycles reinforce each other [10, 77, 80]. This suggests that the three human semilunar and lunar rhythms are controlled by the same lunar clock.

Perhaps some words about the putative origin of the human lunar rhythms should be added. Ethnologists have extensively studied the anthropology of menstruation by investigating rituals and customs in ancient cultures from all over the world [88, 89]. From these studies the consistent image emerges that each ancient culture utilized lunar calendars, in which the female menstrual rhythm played a major role. The lunar month was divided into periods of sexual abstinence, which coincided with women’s menstruation and men’s hunting, as well as periods during which the meat of the hunted animals was prepared, protein-rich meals were consumed, and sexual activities took place. Hunting large animals required not only a concerted action of all men but also long hunting excursions that extended into the night. The latter was only possible during the waxing moon, because only at this time is the Moon already up in the sky in the evening. In most cultures, men were back home around the full Moon, which coincided with the fertile phase of the women that menstruated at new Moon. However, depending on the culture, there are also reports suggesting that women’s fertile phase was during the darkest nights before the new Moon and that men went hunting afterward, returning at the full Moon and preparing the game during the waning Moon phase. This accurately reflects the semilunar nature of the menstruation cycle observed today, in which menstruation can occur around the new or the full Moon even in the same woman (Fig. 3). Interestingly, this observation also fits with the strict lunar ritual (the “Bondo”) that young, sexually mature women of the Temne people in Sierra Leone, West Africa, had to follow during previous centuries before they were allowed to meet their husbands [90]. During the “Bondo” ritual, which could last up to a year, the young women were isolated from the rest of the tribe to live according to the Moon and determine their most fertile phases. In accordance with these phases, they were released to their husbands either before the full Moon or before the new Moon.

In summary, the presence of semilunar and lunar rhythms in algae, cnidarians, mollusks, crustaceans, insects, fish, amphibians, reptiles, birds, and mammals—including humans—suggest that they are not a marine relic, but rather a persistent evolutionary trait across the entire tree of life.

### Evidence for the existence of endogenous circasemilunar and lunar clocks in animals

Despite the many descriptions of effects of the lunar cycle on organisms, it is not clear whether all organisms possess circalunar clocks that anticipate the lunar cycle and fulfill the criteria for an endogenous clock. Several of the observed effects could simply be a direct response to moonlight. Only very few lunar rhythms meet some of the five criteria mentioned in the beginning that need to be fulfilled by a circalunar clock, and in most cases only the first two criteria—a capacity to free-run and a capacity for entrainment—have been demonstrated. The first experimental proof of an endogenous circalunar clock came in 1960 for the marine annelid *Platynereis dumerilii* [40], followed by the brown alga *Dictyota dichotoma* [91] and the marine midge *Clunio marinus* [15]. To date free-running and entrainable circalunar or circasemilunar clocks have been demonstrated in about two dozen species (summarized in [17]). The phenomenon of temperature compensation has only been demonstrated in the annelid *Typosyllis prolifera* [92], the midges *Clunio tsushimensis* [93] and *Pontomyia oceana* [94], and the killifish *Fundulus grandis* [22]. Phase-shifting of the circalunar clock by light pulses have been demonstrated in *Syllis prolifera* [95], *Clunio tsushimensis* [17], and *Platynereis dumerilii* [13]. The demonstration of a limited entrainment range as well as relative coordination at the limits of entrainment is very difficult to achieve and has hence not been performed in these population rhythms of long-term development or reproduction.

The question remains whether the observed temperature-compensated free-running rhythms that can be entrained and phase shifted by light are evidence enough for a circalunar clock. There are alternative hypotheses. The first one was proposed by Erwin Bünning and called the beat hypothesis [91, 96]. It is based on the simultaneous existence of circadian and circatidal clocks as has been shown for intertidal crustaceans [97–101] and grass puffer fish [102]. The superposition of these two clocks will lead to a phase overlap of the ~ 24 h and ~ 12.4 h rhythms every ~ 15 days, which may produce a semilunar rhythm. An alternative hypothesis in organisms that possess no circatidal clock is that a circa(semi)lunar clock relies on a counter mechanism counting ~ 30 or ~ 15 circadian cycles. In this case the circalunar clock would have the same properties as the circadian clock and would just run ~ 30 times slower (or ~ 15 times slower in case of semilunar clocks). Like the circadian clock it will be entrained to 29.52 days by light; but in contrast to the circadian clock the entraining light is not the daily light-dark cycle but the monthly moonlight cycle. Through clever experiments, Bünning and Müller [91] were able to show that brown algae (*Dictyota dichotoma*) use the beat mechanism for timing their semilunar rhythm of spawning. The researchers altered the period of the circadian clock by entraining the algae to light-dark cycles with a Zeitgeber period of 23.5 h instead of 24 h. This had no effect on the circatidal clock, as it does not respond to light. An independent semilunar clock should also remain unaffected by this manipulation; but if the semilunar clock counts the number of days of the circadian clock, it should now have a period that is approximately 8 h shorter (15 × 24 h = *360 h*; 15 × 23.5 h = *352.5 h*; difference 7.5 h). However, if the semilunar clock depends on the interaction between the circadian and circatidal clock according to the beat mechanism, it should have a period that is 2 to 3 days shorter; and this was precisely the case [91, 96]. Similar experiments showed later that the crustacean *Scyphax ornatus* also employs the beat mechanism [103], while the marine midges *Pontomyia oceana* and *Clunio marinus* rely on counting circadian cycles [104, 105]. Different experiments employing molecular methods suggest that the beat mechanism appears also to be used by the crustaceans, *Eurydice pulchra* and *Parhyale hawaiensis* [100, 106] as well as the grass puffer fish, *Takifugu alboplumbeus* [102].

Evidence for a dedicated circalunar oscillator is so far only available for the annelid *Platynereis dumerilii*. Hauenschild [40] performed the same experiment as Bünning and Müller [91] and showed that its circalunar clock is independent of the circadian clock, because it persisted with unchanged period length under daily Zeitgeber cycles of 23.25 h [40]. Furthermore, it continued even after chemical inhibition of the circadian clock [107]. Consequently, it did not rely on the beat or the counting mechanism.

In summary, the aforementioned studies show that the different organisms use different mechanisms for timing circa(semi)lunar rhythms.

### Evidence for the existence of an endogenous circalunar clock in humans

In humans, the persistence of a free-running circalunar clock under constant conditions is even more difficult to test than in animals, because human beings cannot be kept in isolation under such conditions for several months (remember in one year a circalunar clock will only show circa 12 cycles). Furthermore, the criterion of temperature compensation cannot be tested in humans as they are homoiothermic and keep their body temperature constant. Nevertheless, there is some evidence of a free-running circalunar clock that entrains to the lunar cycles in humans, on which we will elaborate in the following.

The first reliable evidence for a circalunar clock that continues in the absence of Zeitgebers comes from the study of Cajochen et al. [78] on human sleep. In this study, the authors retrospectively analyzed the sleep of 33 volunteers that had been monitored in the sleep laboratory between June 17, 2000, and December 2, 2003. In the sleep laboratory, the volunteers could not see the moon, which excludes direct responses to moonlight. Furthermore, neither the volunteers nor the investigators were aware of the a-posteriori analysis of the sleep data relative to lunar phase, which excludes an influence of expectations. Nevertheless, the researchers found that around the full moon, delta activity in the electroencephalogram decreased by 30% during deep sleep, time to fall asleep increased by 5 min, and total sleep duration was reduced by 20 min. These changes were associated with a decrease in subjective sleep quality and diminished endogenous melatonin levels. The authors have been very careful with drawing conclusions, but the most likely interpretation of these findings is that a circalunar clock that modulates sleep does continue in a laboratory study without lunar time cues.

More direct evidence for a human circalunar clock comes from longitudinal studies of the lunar rhythms in mood and menstruation in individual humans [56]. This is because their circalunar nature is already visible in the presence of Zeitgeber cycles, under which they free-run with their endogenous period and are only intermittently synchronous with the lunar cycle. Therefore, the question arises whether this intermittent synchrony relies on real entrainment or happens just by chance. The menstrual cycle and partly also the mood cycles provide decisive evidence that this is genuine entrainment as explained in the following.

**Fig. 4:**
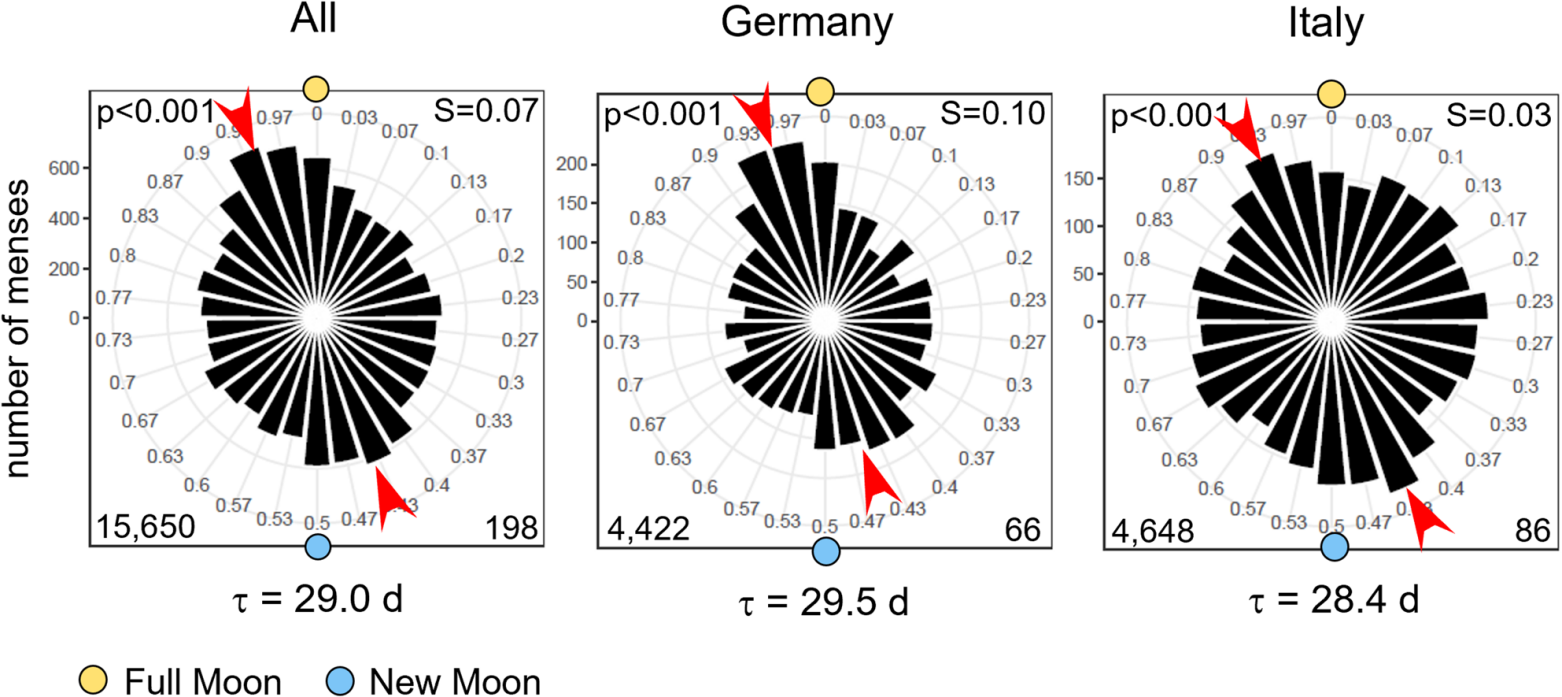
Circular plots combining all 15,650 menses onsets recorded by 198 women (left), the 4,422 menses of the 66 German women (middle) and the 4,648 menses of the 86 Italian women (right). Cycle lengths that were close to the period of the synodic month were one day shorter in Italian women than in German women. The German women had a more peaked distribution of menses onsets than the Italian women, and the phases of the bimodal peaks occurred about 1.5 days before full and new Moon, respectively (red arrowheads). The peak phases of the Italian women occurred about 2 days before the full and new Moon, which is an expected consequence of their shorter cycle length. The peak phases of all the women were in between those of the German and Italian women (data from [10]). The cause for the shorter cycle length of Italian women in comparison to German women might lie in a higher exposure to nocturnal light, as is discussed in [10]. Most of the Italian women who participated in the study were from northern Italy, where light pollution is significantly higher on average than in Germany, and an earlier study has shown that light during the night shortens the period of the menstruation cycle [108]

**Fig. 5:**
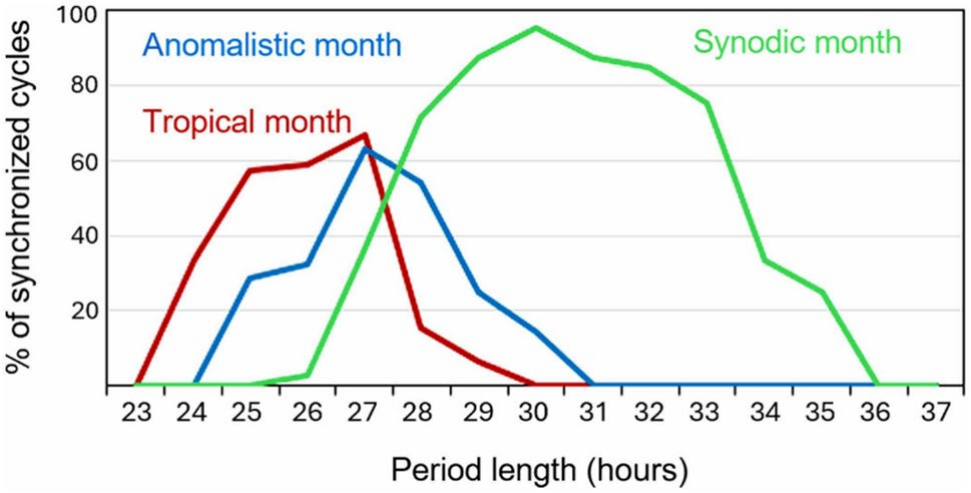
Ranges of entrainment of menstrual cycles to the synodic (29.53d), anomalistic (27.55d), and tropical months (27.32d). For each woman and for each episode with a consistent free-running period, it was determined whether or not menstruation occurred synchronously with one of the three lunar months in at least four consecutive cycles (yes or no). This synchrony could occur before or after episodes with a consistent free-running period. The number of positive events (“yes”) was then expressed as a percentage of all episodes examined and plotted against the free-running period (from [10]

1. In real entrainment, the endogenous clock maintains a specific phase relationship with the Zeitgeber cycle. This is the case for the menstrual and mood cycles, in which menstruations or mania happened either around the full or the new Moon (Figs. 2a and 3) [9, 10, 77]. In the case of menstruation this was not only true for individual women but also on the population level (Fig. 4).
2. The phase relationship of the endogenous clock depends on the period of the free-running rhythm: short-period clocks have an earlier phase relationship to the Zeitgeber than long-period clocks. Again, this was found for the menstrual cycle on the level of individuals and more impressively also on the population level [9, 10]. On average, Italian women had a free-running period that was 1 day shorter than that of German women, and their menstruations peaked 2 days before the full or new moon, while those of the German women peaked 1.5 days before the full or new moon, respectively (Fig. 4).
3. An endogenous clock has a limited range of entrainment. As mentioned above, synchronicity between the lunar cycle and the menstrual cycle was found to occur primarily when the latter lasted approximately 29.53 days [81, 82]. This already suggests that the menstrual cycle cannot entrain to the lunar cycle if its endogenous period is too far away from that of the lunar cycle. It can also explain, why the distribution of menses onsets was less peaked in Italian women, who had an average cycle length of 28.4 days than in German women with a cycle length of 29.5 days (Fig. 4). We have conducted a detailed analysis of the probability of entrainment depending on the free-running period of the cycle before or after a phase of entrainment (for entrainment at least 4 consecutive menstrual events had to occur around the full moon or new moon) [10]. We found that the menstrual cycle had indeed a limited range of entrainment. Cycles free-running with a period longer than 36 days or shorter than 25 days never entrained to the full-new Moon cycle (the synodic month) (Fig. 5). However, cycles with shorter periods could entrain to the anomalistic (27.55 days) or the tropical (27.32 days) month - again with a limited range of entrainment (Fig. 5). Indeed, we found that individual young women were most likely to become entrained to the synodic month. Later, after the endogenous period of their menstrual cycle shortened with age, they intermittently became entrained to the tropical month (Fig. 3a), or to the anomalistic month [9].
4. At the limits of its entrainment, an endogenous clock can show relative coordination with the Zeitgeber cycle. Again, this was shown for the menstruation cycle and can be seen in Fig. 3a.

Altogether, the described phenomena can be hardly explained as merely direct responses to the lunar cycle, they are rather in favor of an endogenous lunar clock in humans that intermittingly entrains to the lunar cycles. Ecochard et al. [109] who analyzed the menstrual cycles of 3024 women that were documented in a large database collected in the last century came to a similar conclusion. The authors investigated the length of successive menstruation cycles and determined whether these are correlated with each other. They found that the duration of a cycle depends on the duration of the previous cycles, meaning that a cycle with longer duration is usually followed by a cycle with shorter duration. This is reminiscent of an endogenous timing system that tries to keep the period length constant [109].

The question remains whether the endogenous lunar clock of humans could rely on a counter or a beat mechanism as found in certain midges and crustaceans [17]. We can clearly exclude the counter mechanism, because, in contrast to the putative circalunar clock, the circadian clock is well entrained to the 24-hour Zeitgeber cycles. If the lunar clock just counts ~ 30 cycles of the circadian clock, it would always be nicely synchronized to the synodic month, and it is clearly not. At first glance, a beat mechanism appears although unlikely, because it depends on the presence of a circatidal clock in addition to a circadian clock, and it is not clear why humans should possess a circatidal clock. However, humans have bimodal rhythms (rhythms that peak twice a day), and recent studies reported that humans have retained genes that control the circatidal rhythms of animals living in the intertidal zone throughout their evolution [110, 111]. In addition, a recent study found tidal rhythms in cardiovascular variables in humans [112]. Therefore, the human circa(semi)lunar clock may work through the beat mechanism (see also discussion in [78]). Nevertheless, the relative coordination of menstrual cycles with the synodic month is not easily explained by the beat mechanism, which assumes that the circatidal clock does not respond to light, as light is not a very good indicator of tides [91].

Perhaps the most likely explanation is that humans possess a real circalunar clock. In contrast to the circadian clock, the circalunar clock lost its stable entrainment to the lunar cycle (for putative reasons see below). Nevertheless, there are other striking parallels between the human circadian and circalunar clocks. The periods of both clocks are strongly influenced by the external factor light and by internal hormonal factors. Light has a strong influence on the free-running periods of both clocks. Constant light slows down the circadian clock, and nocturnal light that is given in the first half of the night delays the phase of the entrained circadian clock [113]. No constant light experiments have been performed for the human circalunar clock, but women who were exposed to light in the night shortened the period of their menstruation cycle [9, 10, 108]. The hormonal state appears also to influence the circadian and circalunar clocks. Roenneberg and his colleagues [114] were the first to demonstrate that the circadian chronotype shifts to later phases during puberty and then, with increasing age, becomes continuously earlier again, a phenomenon that has been confirmed by later studies and appears to be related to age-dependent changes in sex hormones [115–117]. We and others have shown that the period of the menstrual cycle shortens with age and again hormonal changes appear to be responsible for this [9, 10, 118, 119]. These parallels to circadian clocks support our hypothesis that humans indeed possess a circalunar clock.

### Putative physiological basis of circa(semi)lunar clocks

The physiological basis of the mammalian circa(semi)lunar clock is unknown, but it may share the light-input pathways with the circadian clock in the suprachiasmatic nuclei (SCN), which are quite light-sensitive and can respond to moonlight [120, 121]. Light is perceived by rods, cones and melanopsin-positive ganglion cells of the eye and transmitted by the ganglion cells via the retino-hypothalamic tract to neurons in the ventral part of the SCN, which express the neuropeptide vasoactive-intestinal polypeptide (VIP) [122–124]. Most interestingly, VIP-expressing neurons project to the medial preoptic area, which is a major integrating center that plays a role in the control of sleep and thermoregulation [125], mood [126], reproduction [127, 128], and many other autonomic functions that are necessary for species survival. As for reproduction, VIP neurons project directly to gonadotropin-releasing hormone (GnRH) neurons in the medial preoptic area, and VIP was shown to excite these neurons in mouse brain slices [123, 129, 130]. A surge of GnRH release provokes the surge in luteinizing hormone (LH) that triggers ovulation, indicating that VIP influences the timing of ovulation. So far, VIP is thought to mainly gate the daily pattern of GnRH-LH release and not to control the infradian estrous cycle, but it is well known that a deficiency in VIP disrupts the estrous cycle [129–131]. Thus, VIP might be necessary to transmit daily moonlight information to a putative circa(semi)lunar clock in the medial preoptic area to control a normal estrous cycle. Alternatively, a circatidal clock could tick in the medial preoptic area, as was shown by Zhu et al. [110] in several other mammalian tissues, and this circatidal clock could cooperate with the circadian oscillations of VIP to generate (semi)lunar rhythms in the estrous cycle via the beat mechanism. Without VIP this (semi)lunar rhythm would be disturbed. At least in fish, it was shown that the expression of many genes oscillates in a circatidal manner in the hypothalamus and the pituitary [24], while several genes in the pineal gland, which appears to contain the circadian master clock, oscillate in a circadian manner and are entrained to the 24 h light-dark cycle if present [102]. These possible circatidal and circadian clocks may then cooperate to drive circasemilunar rhythms via the beat mechanism [102]. Similarly, circadian and circatidal clocks are interconnected in invertebrates, such as intertidal crustaceans. Here, the two clocks utilize similar molecular clock components yet generate circadian and circatidal oscillations in different brain regions [100, 101]. It remains to be seen which mechanism operates in the mammalian brain.

In any case, it is still unclear how gravitational cycles might influence the circatidal or circa(semi)lunar clock in animals. The effects of lunar-solar gravitational forces on such small organisms are negligible, and there are no known sensory organs capable of detecting them. It is therefore more likely that these forces are perceived indirectly through changes in other physical parameters influenced by the lunar cycle. Changes in geomagnetic fields, which may be detected via cryptochromes, are likely candidates. There are various cryptochromes, and some of them respond not only to changes in light but also to small changes in the magnetic field [132]. In *Platynereis dumerilii*, Cryptochrome is necessary to distinguish moonlight from sunlight [13, 133] and one can easily imagine that small changes in the geomagnetic field can enhance the effects of moonlight. The Moon’s influence on the Earth’s magnetic field is also discussed as possible cause of (semi)lunar oscillations in the fertility of farm animals [62].

### Why should humans possess a circa(semi)lunar clock?

Unlike aquatic organisms with external fertilization or a very short adult lifespan, the fitness of humans (and probably other terrestrial animals with long lifespans) does not seem to depend on a circalunar clock that predicts the phase of the Moon. Nevertheless, as mentioned earlier, humans have probably benefited since ancient times from being active on moonlit nights, either to take advantage of longer hunting, foraging or social opportunities [89, 134]. Humans who avoided outdoor activities on dark new Moon nights and instead used this time for reproduction may have had a fitness advantage. Others may have taken advantage of the increased social activity around the full Moon for reproduction, which may also have led to a fitness advantage. Our studies showed that menstruation occurred more frequently around the full Moon and new Moon, suggesting that ovulation clustered around the new Moon and full Moon in populations of women [9, 10]. Thus, both reproductive strategies appear successful.

It would be extremely interesting to investigate whether men exhibit similar lunar rhythms in their fertility. This seems likely, as the onset of mania during full moon and new moon is accompanied by a dramatic increase in libido and sexual behavior in both women and men [75]. This not only indicates that mood cycles and cycles in the reproductive neuroendocrine system are linked but also strongly suggests that libido may also be increased in people with normal lunar mood cycles that do not lead to mania. Future studies are needed to investigate this in detail and determine whether these lunar rhythms also influence other aspects of human physiology and health, as well as to elucidate the mechanisms by which such effects may occur.

### Consequences of artificial light at night on the human circalunar clock

As mentioned earlier, the circalunar clock has obviously lost its stable entrainment to the lunar cycle, but we do not know when this happened. Unfortunately, there are no reliable records of mood and menstrual cycles prior to the invention of electric light, and even older records from the middle of the last century found only a weak entrainment to the synodic month [9, 75, 109].

It is possible that the stable entrainment of behavior to the lunar cycle was gradually lost as human societies transitioned from hunter-gatherer lifestyles to modern agricultural and industrial societies. At the same time, lunar calendars were replaced by solar calendars, since it was no longer necessary for human societies to align themselves perfectly with the lunar cycle in order to survive, and solar calendars were more practical, especially at higher latitudes.

Nevertheless, it is very likely that increased exposure to artificial light is one of the factors that has reduced the likelihood that our circalunar clock could entrain to the luminous cycles of the moon. At the population level, the weak entrainment of menstrual cycles to the synodic month was lost after the introduction of powerful light-emitting diodes (LEDs) and the increasing use of smartphones and other light-emitting devices [10]. In addition, humans isolate themselves from moonlight with architectural barriers. What has remained over the centuries is a weak entrainment of the circalunar clock to the gravitational cycles of the moon, and this applies to mood, sleep, and menstrual cycles [9, 10, 75, 80]. Rodriguez Ferrante et al. [80] investigated sleep onset by analyzing longitudinal actigraphic recordings from urban cohorts in Seattle, indigenous Toba/Qom communities in Argentina, and captive titi monkeys with attenuated access to natural light. They found that sleep onset was delayed in all three populations around periods of maximal gravimetric forces of the Moon, regardless of whether the maxima occurred during the full or new Moon. However, in the Toba/Qom communities, which have very limited access to electric lighting, sleep was additionally influenced by moonlight and was shorter around the full Moon. Menstrual cycles achieved the strongest synchronization with the full/new Moon cycle in January during perihelion (Fig. 1a), when the gravimetric forces of the sun are added to those of the moon, and this was even true in recent times [10]. Together, these results suggest that the brightness and gravimetric cycles of the moon synergistically entrain the circalunar clock, with the brightness cycles dominating, but that the gravimetric cycles take over when artificial light at night obscures the full Moon/new Moon cycles. However, gravimetric cycles are very weak Zeitgebers. They appear even weaker than moonlight cycles.

Artificial light at night not only obscures natural moonlight cycles but appears also to shorten the period of the circalunar clock (see above). This shortening, in turn, reduces the probability of entrainment to the lunar cycle, as ongoing entrainment is only possible if the period of the circalunar clock is close to that of the lunar cycle. Because menstrual cycle length appears to be an age-dependent marker of female fertility [135], entrainment of the circalunar clock might prove to be relevant not only to human physiology and behavior but also to fertility and contraception.

## Conclusions

Lunar and semilunar rhythms are widespread in the animal kingdom. Although there are several putative mechanisms that can lead to (semi)lunar rhythms, some animals, including humans, appear to have circalunar clocks that control reproduction, physiology and behavior (in humans in particular sleep/wake activity and mood). The human circalunar clock is only temporarily entrained to the 29.53-day synodic month, the 27.55-day anomalistic month, or to the 27.31-day tropical month. The primary Zeitgebers are moonlight and, secondarily, gravimetric lunar cycles. Entrainment to the lunar cycles is generally weak and has decreased significantly in recent times, most likely because our modern lifestyle with increased artificial light at night obscures the natural moonlight cycles. At present, it is difficult to say what consequences the loss of apparent synchronicity with the lunar cycle has for our health. Nevertheless, it is possible that this is one of the many factors that influence our fertility, which has declined in advanced societies in recent decades. While humans’ exposure to moonlight in the modern environment has decreased, their exposure to lunar gravimetric cycles continues unabated. Lunar cycles in gravity may account for the persistence of lunar cycles in sleep in the modern environments. Cycles in sleep are relevant to human performance and well-being, and to bipolar and other disorders that are sensitive to alterations in sleep. In future research, the question of whether changes in gravity or other factors play a role in the entrainment of biological rhythms to lunar cycles could be addressed by blocking individuals’ exposure to moonlight. Conversely, experiments could determine whether exposure of modern humans to cycles of artificial moonlight could restore lunar cycles in biology and behavior, and whether such interventions are beneficial to human health and disease. The biological mechanisms that mediate humans’ responses to lunar cycles are unknown. A possible clue to mechanism lies in the fact that lunar cycles in some cases of bipolar disorder depend on continuous administration of antidepressant medications [78]. Known or unknown mechanisms of antidepressants and other pharmacological agents could elucidate mechanisms of humans’ responses to the Moon.

## Data Availability

No datasets were generated or analysed during the current study.
